# High-Hall-Mobility Al-Doped ZnO Films Having Textured Polycrystalline Structure with a Well-Defined (0001) Orientation

**DOI:** 10.1186/s11671-016-1535-1

**Published:** 2016-06-30

**Authors:** Junichi Nomoto, Hisao Makino, Tetsuya Yamamoto

**Affiliations:** Research Institute, Kochi University of Technology, 185 Miyanokuchi, Tosayamada-cho, Kami-shi, Kochi 782-8502 Japan

**Keywords:** Carrier transport, Transparent conducting oxide, X-ray diffraction, Al-doped ZnO, Ga-doped ZnO, Magnetron sputtering, Ion plating

## Abstract

Five hundred-nanometer-thick ZnO-based textured polycrystalline films consisting of 490-nm-thick Al-doped ZnO (AZO) films deposited on 10-nm-thick Ga-doped ZnO (GZO) films exhibited a high Hall mobility (*μ*_H_) of 50.1 cm^2^/Vs with a carrier concentration (*N*) of 2.55 × 10^20^ cm^−3^. Firstly, the GZO films were prepared on glass substrates by ion plating with dc arc discharge, and the AZO films were then deposited on the GZO films by direct current magnetron sputtering (DC-MS). The GZO interface layers with a preferential *c*-axis orientation play a critical role in producing AZO films with texture development of a well-defined (0001) orientation, whereas 500-nm-thick AZO films deposited by only DC-MS showed a mixture of the *c-*plane and the other plane orientation, to exhibit a *μ*_H_ of 38.7 cm^2^/Vs with an *N* of 2.22 × 10^20^ cm^−3^.

## Background

We demonstrate a nanoscale material design to achieve high-Hall-mobility Al-doped ZnO (AZO) textured polycrystalline films with a well-defined (0001) orientation. The key factor is to enhance intragrain carrier mobility together with a substantial reduction of the contribution of grain boundary (GB) scattering to carrier transport due to a high degree of *c*-axis alignment between columnar grains. AZO films have recently been focused on as an alternative to tin-doped indium oxide (In_2_O_3_:Sn) and fluorine-doped tin oxide (SnO_2_:F) films for use as the transparent electrodes of flat panel displays and in the window layers of solar cells [1–4]. In our previous work [5], we investigated the characteristics of AZO films deposited by direct current magnetron sputtering (DC-MS), radio frequency (RF)-MS, and RF-superimposed DC-MS (RF/DC-MS) with a systematic variation of the power ratio of DC to RF to clarify key factors that determine the carrier transport. We used sintered oxide argets with an Al_2_O_3_ content of 2.0 wt.%. The AZO films deposited by DC-MS had a high carrier concentration (*N*), however, showing poor *c*-axis alignment between the columnar grains of the polycrystalline structure with textures having a mixed orientation of atomically closely packed (0001) and $\left(10\overline{1}1\right)$ planes, whereas AZO films with a low *N* deposited by RF-MS exhibited a texture consisting of well-aligned columnar grains that showed a preferential *c*-axis orientation perpendicular to the substrate. Analysis of the relationship between the carrier transport and the orientation distribution of AZO films by various MS techniques showed that the presence of the $\left(10\overline{1}1\right)$ orientation texture can increase the contribution of GB scattering to carrier transport, resulting in a reduced Hall mobility (*μ*_H_). The above contribution was defined as the ratio of the optical mobility (*μ*_opt_) corresponding to intragrain carrier mobility to the carrier mobility at the GBs (*μ*_GB_), *μ*_opt_/*μ*_GB_ = (*μ*_opt_ − *μ*_H_)/*μ*_H_ [5]. *μ*_opt_ was calculated on the basis of the Drude theory with the Tauc-Lorentz model [6–10] using optical data such as optical transmittance and reflectance. From those findings, an issue to be resolved to achieve high-*μ*_H_ AZO films with low electrical resistivity (*ρ*) is to develop a deposition technology to realize AZO films with a well-defined single (0001) orientation by DC-MS.

In this work, on the basis of many reports on controlling the crystallinity and/or surface morphology of AZO films in the early growth stages [11–18], we propose a resolution to the issue; a critical layer (CL) made from 10-nm-thick Ga-doped ZnO (GZO) films with a preferential *c*-axis orientation normal to the substrate as an interface layer between thicker AZO films by DC-MS and substrates. The CL comprises GZO films deposited by ion plating (IP) with dc arc discharge. The IP technique enables us with the optimization of the energy of the incident particles from the target to the substrate surface, with a high plasma density in the order of 10^12^/cm^3^ resulting in high ionization rates of Zn, Ga, and O atoms, to promote the formation of the columnar grains with the highly preferential *c*-axis orientation owing to the well-defined (0001) orientation in the entire GZO films and to enhance high lateral diffusion of the above species leading to the films exhibiting the flat surface [19, 20]. The CLs play a critical role in achieving the AZO films having a textured polycrystalline structure with a well-defined single (0001) orientation.

It also should be noted that for wide applications of highly transparent conducting AZO films, taking into account the fact that *N* in the range from 4× to 6 × 10^20^ cm^−3^ gives rise to the reduction in the optical transmittance from the visible (VIS) to near-infrared (NIR) wavelength region due to the free carrier absorption, the magnitude of *N* should be in the range from 2× to 3 × 10^20^ cm^−3^. In the present work, we, thus, used sintered oxide targets with an Al_2_O_3_ content of 0.5 wt.%. In such case, high *μ*_H_ is essential for use as highly transparent conducting films. We demonstrate that the use of CLs is an effective way to achieve the above films.

We investigated the effects of the improved orientation distribution due to the use of CLs on the electrical and optical properties to clarify a limiting factor of carrier transport of AZO films by DC-MS.

## Methods

### Film Deposition

We deposited 500-nm-thick AZO films on glass substrates (Corning Eagle XG) or 490-nm-thick AZO films on 10-nm-thick CLs prepared on the glass substrates at a substrate temperature of 200 °C by DC-MS with a power of 200 W. We used an MS apparatus (ULVAC CS-L) to deposit the AZO films [5]. The sintered oxide targets (Toshima Manufacturing Corp.) were high-density sintered circular AZO targets (diameter: 80 mm) with an Al_2_O_3_ content of 0.5 wt.%. We deposited 10-nm-thick CLs made from GZO films on the glass substrates using IP apparatus (Sumitomo Heavy Industries, Ltd.) with a dc arc discharge current of 150 A. We introduced oxygen (O_2_) gas with a flow rate of 10 sccm into the chamber to control the density of oxygen-related point defects such as oxygen vacancies and interstitials in the film. The evaporation source (HAKUSUI Tech. SKY-Z) for the deposition of CLs was sintered ceramic ZnO (99.99 % purity) containing 4.0 wt.% Ga_2_O_3_ (99.9 % purity) [10, 20].

### Characterization

The film thickness was measured using a surface profilometer (KLA Tencor, Alpha-Step IQ). *N*, *μ*_H_, and *ρ* were determined by Hall effect measurements (Nanometrics, HL5500PC) at a room temperature using the van der Pauw method. Indium paste was used as an ohmic contact electrode between measuring styluses and surface of AZO films.

The out-of-plane *θ*/2*θ* X-ray diffraction (XRD) pattern and out-of-plane rocking curve were obtained. For a comprehensive analysis of the texture evolution, we carried out out-of-plane grazing-incidence XRD (GIXRD) measurements, where the X-ray incident beam angle (*ω*) was fixed at 0.35° and only the 2*θ* axis was scanned [21–24]. The crystal structure of the films was characterized by out-of-plane wide-range reciprocal space maps (RSMs) obtained using the SmartLab high-resolution XRD system (Rigaku corp.) equipped with a PILATUS 100K/R two-dimensional (2D) X-ray detector using Cu-K *ā* (wavelength *λ* = 0.15418 nm) radiation [25, 26]. The optical properties were measured using a spectrophotometer (Hitachi, U-4100) and a spectroscopic ellipsometer (SE; J.A. Woollam, M-2000DI). The optical transmittance (*T*) and reflectance (*R*) spectra of the ZnO-based films in the wavelength (*λ*) range from 200 to 2400 nm were obtained using a spectrophotometer with an incident angle of light of 5°. The ellipsometric data (*Ψ* and *Δ*) [10] were acquired in the wavelength range from 300 to 1700 nm at incident angles of 55° to 75° in 5° step.

## Results and Discussion

**Fig. 1:**
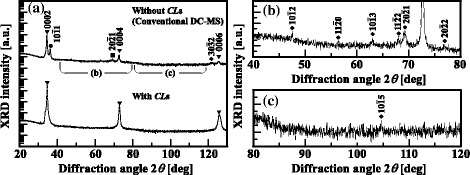
Out-of-plane *θ*/2*θ* XRD patterns of AZO films (**a**) without and with CLs and of CL-free AZO films in the 2*θ* ranges (**b**) from 40° to 80° and (**c**) from 80° to 120°

Figure 1(a) shows out-of-plane *θ*/2*θ* XRD patterns for AZO films without and with CLs. The analysis of the out-of-plane *θ*/2*θ* XRD patterns of the two different types of AZO films shows that the intensity of wurtzite ZnO 0002 peak is much higher than those of the other peaks. For AZO films without CLs, several peaks except for the 0002 peak were clearly observed. The out-of-plane *θ*/2*θ* XRD patterns of AZO films without CLs in the 2*θ* ranges from 40° to 80° and from 80° to 120° are shown in Fig. 1(b, c), respectively: the peaks of the $10\overline{1}1$, $10\overline{1}2$, $10\overline{1}3$, $10\overline{1}5$, $11\overline{2}0$, $11\overline{2}2$, $20\overline{2}1$, $20\overline{2}2$, and $30\overline{3}2$ reflections together with those of the 0002, 0004, and 0006 reflections were observed. On the other hand, for AZO films with CLs, we found high intensities of the 0002, 0004, and 0006 reflections compared with those of the CL-free AZO films. Note that unlike CL-free AZO films, as shown in Fig. 1(a), no other peaks have been observed for AZO films with CLs, as shown in Fig. 1(b, c).

**Fig. 2:**
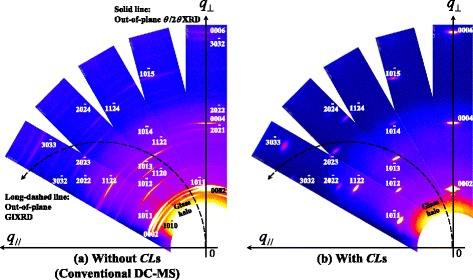
Wide-range reciprocal space maps (RSMs) of 500-nm-thick AZO films **a** without and **b** with CLs

To investigate what causes the significant difference in the above behavior of the reflections, as shown in Fig. 1(a–c), between AZO films with and without CLs, we examined the location of the center of gravity of the diffraction peaks obtained by wide-range RSMs. Figure 2 shows the following results: *q*_//_ and *q*_⊥_ represent the coordinates of the reciprocal space (*q =* 1/*d*_*hkil*_ = 2sin*θ*/*λ*, *θ* and *λ* are the incident angle and the wavelength of the X-rays, respectively); *q*_//_ is parallel to the surface, and *q*_⊥_ is perpendicular to the surface. The solid and long-dashed directional lines correspond to orbitals for the out-of-plane *θ*/2*θ* XRD and GIXRD scans, respectively. For the AZO films without CLs, as shown in Fig. 2a, the analysis of the data obtained by RSMs showed the peaks of the $10\overline{1}1$, $10\overline{1}2$, $10\overline{1}3$, $10\overline{1}5$, $11\overline{2}0$, $11\overline{2}2$, $20\overline{2}1$, $20\overline{2}2$, and $30\overline{3}2$ reflections together with those of the 0002, 0004, and 0006 reflections. Note that the center of gravity of the peaks of the symmetrical $10\overline{1}1$, $20\overline{2}1$_,_$20\overline{2}2$, and $30\overline{3}2$ reflections was observed to be located approximately on a vertical line in the RSMs. In addition, the center of gravity of the peaks of the other reflections such as $10\overline{1}2$, $10\overline{1}3$, $10\overline{1}5$, $11\overline{2}0$, and $11\overline{2}2$ reflections were located on an asymmetrical zones in RSMs. This proves that the $\left(10\overline{1}1\right)$, $\left(20\overline{2}1\right)$, and $\left(30\overline{3}2\right)$ planes of the AZO films without CLs lie approximately parallel to a substrate surface. On the other hand, the polycrystalline AZO films with CLs, as shown in Fig. 2b, show a distinct feature: we found that only {0001} family of planes was parallel to the substrate surface. This indicates that the AZO films with CLs exhibit a high degree of *c*-axis alignment between columnar grains: we confirmed a small full width at half maximum of the *ω* rocking curves (FWHM*ω*) for the 0002 peak of 1.81° compared with those, FWHM*ω* >2, for GZO films deposited by IP with dc arc discharge in our previous work [10].

Next, we investigated the effects of CLs on the electrical properties of AZO films with CLs. Table 1 summarizes the results of Hall effect measurements at a room temperature. In this study, taking into account the fact that the thickness of CLs was 10 nm, which was very thin compared with the total thickness of the samples of 500 nm, the discussion below is based on a single-layer model. We obtained the following results in the experiments: the AZO films with CLs showed a lower *ρ* of 4.89 × 10^−4^ Ωcm with a *μ*_H_ of 50.1 cm^2^/Vs and an *N* of 2.55 × 10^20^ cm^−3^, whereas CL-free AZO films exhibited a higher *ρ* of 7.26 × 10^−4^ Ωcm with a *μ*_H_ of 38.7 cm^2^/Vs and an *N* of 2.22 × 10^20^ cm^−3^. The cause of the drastic reduction in *ρ* of the AZO films with CLs compared with that of the CL-free AZO films is an enhancement in both *μ*_H_ and *N*, considering the inversely proportional relationship between *ρ* and the product of *μ*_H_ and *N*. The use of CLs produced a distinct increase in *μ*_H_ for AZO films by 29 % together with an increase in *N* by 15 %. Several investigations on the control of *μ*_H_ for almost same values of *N* have been conducted for the following films: (1) ZnO films codoped with Al and hydrogen deposited by RF-MS [27], (2) AZO films deposited by RF-MS [28] and DC reactive MS [29], and (3) boron (B)-doped ZnO (BZO) deposited by pulsed laser deposition (PLD) [30]. In general, the growth temperature, thickness, and types of the substrates strongly affect the electrical properties of AZO films regardless of the deposition methods. Therefore, we compare the films deposited in this study with 500-nm-thick BZO films deposited on a glass substrate at a temperature of 200 °C by PLD, for which *μ*_H_ and *N* were reported to be 47.2 cm^2^/Vs and 2.97 × 10^20^ cm^−3^, respectively [30]. Considering that the MS and IP techniques have the advantages of growth areas and deposition rates over the PLD technique, the use of nanometer-thick CLs with these deposition methods is very effective for achieving high-*μ*_H_ AZO films not only from academic nanoengineering research but also from industrial engineering viewpoints.

**Table 1:** Electrical resistivity (*ρ*), carrier concentration (*N*)*,* Hall mobility (*μ*_H_), optical mobility (*μ*_opt_), the contribution of grain boundary scattering to carrier transport (*μ*_opt_/*μ*_GB_; carrier mobility at grain boundaries), effective mass of electrons (*m* ^*^), plasma frequency (*ω*_p_), and high-frequency dielectric constant (*ε*_∞_) of AZO films with and without CLs

Structure	*ρ* (Ωcm)	*N* (cm^−3^)	*μ*_H_ (cm^2^/Vs)	*μ*_opt_ (cm^2^/Vs)	*μ*_opt_/*μ*_GB_	*m* ^*^	*ω*_p_ (rad/s)	*ε*_∞_
With CLs	4.89 × 10^−4^	2.55 × 10^20^	50.1	50.1	0.00	0.24	9.32 × 10^14^	3.96
Without CLs	7.26 × 10^−4^	2.22 × 10^20^	38.7	51.1	0.32	0.23	8.83 × 10^14^	3.92

In the following, to obtain a better understanding of the change in *μ*_H_ attributed to the use of the CLs, we calculated *μ*_opt_/*μ*_GB_ on the basis of Matthiessenʼs rule, (*μ*_H_)^−1^ = (*μ*_opt_)^−1^ + (*μ*_GB_)^−1^ [5]. *μ*_opt_ was calculated on the basis of the Drude theory [5–10] using the experimental data for *Ψ* and *Δ* determined by spectroscopic ellipsometry measurements combined with experimental data for *T* and *R* determined by spectrophotometer measurements. To describe the optical response due to free electrons, the dielectric function of AZO films based on the conventional Drude model (*ε*_D_) is expressed by

1 $$ {\varepsilon}_D(E)=-\frac{A_D}{E^2-i{\varGamma}_DE}, $$

where *A*_*D*_ and *Γ*_*D*_ are the oscillator amplitude and broadening parameter, respectively [5–10]. In the Drude theory for free electrons, *Γ*_*D*_ is expressed as

2 $$ {\varGamma}_D=\frac{\hslash e}{m^{*}{\mu}_{\mathrm{opt}}}, $$

where *ℏ* ≡*h*/2π (*h* is Planck’s constant), *e* is the electron charge, and m^*^ is the effective mass of electrons. In this study, we calculated m^*^ by the following process. In the Drude model, the real part of *ε*_D_ (Re*ε*_D_) is given by

3 $$ Re{\varepsilon}_{\mathrm{D}}={\varepsilon}_{\mathrm{D}}^{\prime }={n}^2-{k}^2={\varepsilon}_{\infty}\left(1-\frac{\omega_{\mathrm{p}}^2}{\omega^2+{\omega}_{\mathrm{c}}^2}\right), $$

where *n* and *k* are the refractive index and extinction coefficient, respectively, and *ε*_∞_ is the high-frequency dielectric permittivity due to the bound electrons. In metal-like materials, two characteristic frequencies, the plasma frequency *ω*_p_ and collision frequency *ω*_c_, are completely defined by the free carriers; *ω*_p_ is given by *ω*_*p*_ = ((*e*^*2*^*N*)/(m^*^ε_∞_*ε*_o_))^1/2^, where *ε*_o_ is the free-space dielectric constant and *ω*_c_ is the reciprocal of the relaxation time. In the high-frequency region, where *ω*_c_ in Eq. (3) can be neglected, *ε*_D_*'* = *ε*_∞_ × (1 − (*ω*_p_*/ω*)^2^) can be used as a first approximation. By plotting *ε*_D_*'* versus *ω*^2^, *ω*_p_ and *ε*_∞_ can be determined from the gradient and intercept obtained, respectively. Using these values, m^*^ is calculated from the above expression for *ω*_p_.

Table 1 shows the values of *ω*_p_, *ε*_∞_, and m^*^ obtained by the best fit in the wavelength range from 0.65 to 0.9 *μ*m. *μ*_opt_ calculated using Eq. (2) with *Γ*_*D*_ obtained by the SE analysis, calculated values of m^*^and *μ*_opt_/*μ*_GB_ obtained using Matthiessenʼs rule with *μ*_H_ determined by Hall effect measurements and also tabulated in Table 1. Note that the AZO films with CLs had a *μ*_opt_ of 50.1 cm^2^/Vs together with a *μ*_opt_/*μ*_GB_ of practically zero, leading to no reduction in *μ*_H_ to 50.1 cm^2^/Vs as a result, whereas the CL*-*free AZO films had a *μ*_opt_ of 51.1 cm^2^/Vs similar to that of the AZO films with CLs, and, however, exhibited a large *μ*_opt_/*μ*_GB_ of 0.32, resulting in a substantially reduced *μ*_H_ of 38.7 cm^2^/Vs compared with the above *μ*_opt._ This clearly demonstrated that the texture of a mixture with the (0001) and the other orientations, as shown in Fig. 1(a), gives rise to an increase in the contribution of GB scattering to carrier transport. The CLs play a critical role in substantially reducing the contribution of GB scattering to carrier transport owing to the improved degree of *c*-axis alignment between columnar grains, thereby improving *μ*_H_.

**Fig. 3:**
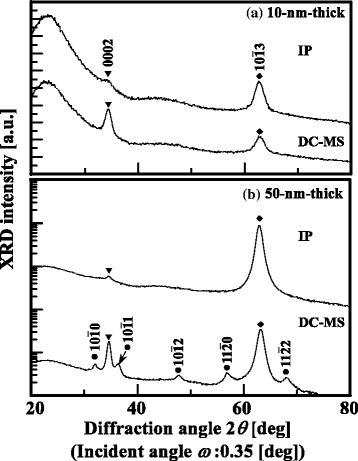
Out-of-plane GIXRD patterns of **a** 10- and **b** 50-nm-thick AZO films deposited by DC-MS and GZO films prepared by IP with dc arc discharge

Then, the evolution of the orientation distribution in the initial stage of film growth should be discussed. Figure 3a shows the out-of-plane GIXRD patterns of 10-nm-thick AZO films deposited on glass substrates by DC-MS or CLs, i.e., 10-nm-thick GZO films deposited on glass substrates by IP with dc arc discharge. Figure 3b shows the out-of-plane GIXRD patterns of 50-nm-thick AZO films deposited on glass substrates by DC-MS and 50-nm-thick GZO films deposited on glass substrates by IP with dc arc discharge. The analysis of the out-of-plane GIXRD patterns [21–24] for two different types of ZnO-based films with a thickness of 10 nm in Fig. 3a shows the following: the DC-MS technique produced films with predominant 0002 and $10\overline{1}3$ peaks, whereas the IP technique generated films showing a $10\overline{1}3$ peak with very high intensity and a 0002 peak with very low intensity, which suggests a well-defined (0001) orientation. Upon increasing the thickness to 50 nm (Fig. 3b), the AZO films deposited by DC-MS clearly exhibited the $10\overline{1}0$, $10\overline{1}1$, $10\overline{1}2$, $11\overline{2}0$, and $11\overline{2}2$ peaks in addition to the 0002 and $10\overline{1}3$ peaks. From Figs. 1(b), 2a, and 3b, it is likely that 10-nm-thick AZO films by DC-MS have a polycrystalline textured structure consisting of some crystallites with a low probability of different orientations from the (0001) orientation. On the other hand, Fig. 3a, b shows that the reflections remained the same, corresponding to the 0002 and $10\overline{1}3$ peaks, for the GZO films with different thicknesses prepared by IP with dc arc discharge. This proves that 10-nm-thick GZO films deposited by IP with dc arc discharge show high crystallinity owing to the well-defined (0001) orientation mentioned above over the entire film.

**Fig. 4:**
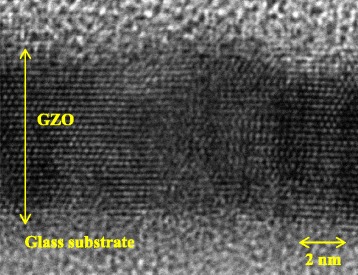
Cross-sectional bright-field TEM image of a 10-nm-thick GZO film deposited on a glass substrate grown by IP with dc arc discharge

Figure 4 shows a cross-sectional bright-field TEM image of a 10-nm-thick GZO film deposited on glass substrates grown by IP with dc arc discharge. Note that lattice fringes were clearly observed for the film. Qualitative analysis of the data obtained by X-ray reflectivity (XRR) measurements by computer simulation suggests the presence of a multilayer; a GZO film with a higher density/an interfacial about 2-nm-thick GZO layer with a lower density directly/the glass substrate. This implies that ZnO deposition is less favorable at the glass substrate surface, hindering the growth of ZnO-based film for the first few layers. The GZO film can be thinner than expected as a result. The GZO films might relax the misfit by distorting their lattice in the interfacial layer. Consequently, the remaining GZO films with a higher density exhibit the (0001) orientation distribution. The XRR-based study on the dependence of the microstructure on the deposition parameters for very thin ZnO films deposited on glass substrates will be reported elsewhere. From the above findings, the use of high crystal quality CLs exhibiting the distinct feature of the evolution of the orientation distribution produces AZO polycrystalline films having a textured structure with a well-defined (0001) orientation subsequently grown on the CLs.

**Fig. 5:**
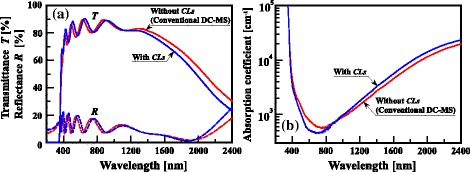
**a** Optical transmittance (*T*) and reflectance (*R*) spectra and **b** absorption coefficient (*α*) spectra of 500-nm-thick AZO films without and with CLs

Next, we demonstrate the variations in *T*, *R*, and absorption coefficient (*α*) of the AZO films without and with CLs. Figure 5a, b shows the *T*, *R*, and *α* of glass substrates with the two different types of AZO films as functions of wavelength (*λ*), respectively. *α* is evaluated using the relation [31, 32]

4 $$ \alpha =\frac{1}{t} \ln \left(\frac{1-R}{T}\right), $$

where *t* is the film thickness of 500 nm. The *α* curve of AZO films with CLs in Fig. 5b exhibits a typical characteristic of transparent conductive films, showing the minimum (*α*_min_) in the VIS region ranging from 400 to 700 nm. The magnitude of *α*_min_ would be limited by the absorption mode from various scattering centers such as ionized donor and acceptor impurities, neutral impurities, phonons, and free carriers. Figure 5b clearly demonstrated that the CLs play an important role in substantially reducing *α* in the VIS region. Considering that the optical absorption in the VIS range is proportional to the number of free carriers and AZO films with CLs had slightly higher *N* than CL-free AZO films as shown in Table 1, this indicates that, besides free carriers, the other scattering centers, such as *n*-type oxygen vacancies (*V*_O_) and/or *n*-type zinc interstitials (Zn_*i*_) and structural defects may also be possibly influencing the absorption source. In our previous work [33], we demonstrated that Ga doping of ZnO films, which leads to an increase in the magnitude of the Madelung energy, enhances the stability of oxygen species in the vicinity of the sites of Ga atoms substituting Zn atoms; some amount of Ga species added to ZnO films removes the ionized point defects such as *V*_O_ and/or Zn_*i*_ by substituting Zn atoms together with strong attractive Coulomb interaction with the O atoms close to the Ga donors and/or by the reaction with Zn_*i*_ thorough repulsive Coulomb interaction between the two different kinds of donors. The two-step deposition process using CLs made from the GZO films can be an effective way to produce AZO films with not only the enhanced preferential *c*-axis orientation but also improved crystallinity as well as the CLs. The reduction of the above scattering centers consisting of the *n-*type intrinsic defects would lead to the reduction of optical absorption of AZO films. The behavior of lowered *α* in the VIS range of AZO films with CLs was observed as a result. In addition, we found that the average *T* (*T*_av_) values in the VIS region of the AZO films without and with CLs were 81.7 and 82.5 %, respectively, as shown in Fig. 5a; the use of CLs causes a slightly increase in *T*_av_ owing to the improved degree of crystallinity. Figure 5b shows that as *λ* is increased towards NIR spectral range from 800 to 2400 nm, AZO films with CLs had high *α* values compared with those of AZO films without CLs at any given *λ*. This indicates the consistency between *α* and *N* of AZO films; the optical absorption in the above spectral range gets influenced by absorption centers, free carriers.

Finally, it should be noted that the CL technique is also very effective for achieving high *μ*_H_ AZO films with thinner films as a result of a successful texture-controlled growth: 200- and 100-nm-thick AZO films with the 10-nm-thick CLs show a *ρ* of 6.91 × 10^−4^ Ωcm with an *N* of 2.25 × 10^20^ cm^−3^ and a *μ*_H_ of 40.2 cm^2^/Vs and a *ρ* of 8.34 × 10^−4^ Ωcm with an *N* of 2.31 × 10^20^ cm^−3^ and a *μ*_H_ of 32.5 cm^2^/Vs, respectively. On the other hand, 200- and 100-nm-thick AZO films without CLs exhibit a *ρ* of 1.18 × 10^−3^ Ωcm with an *N* of 1.82 × 10^20^ cm^−3^ and a *μ*_H_ of 29.2 cm^2^/Vs and a *ρ* of 1.33 × 10^−3^ Ωcm with an *N* of 1.75 × 10^20^ cm^−3^ and a *μ*_H_ of 26.9 cm^2^/Vs, respectively. These results show that the use of CLs for 200- and 100-nm-thick AZO films enhances *N* by 23.7 % together with the improvement of *μ*_H_ by 37.7 % and increases *N* by 32.0 % together with the improved *μ*_H_ by 20.8 %, resulting in a large decrease in *ρ* by 41.2 % and by 37.3 % compared with those for 200- and 100-nm-thick AZO films deposited by a conventional DC-MS, respectively. These findings suggest that the development of the CL technique is expected to help understand the mechanisms limiting carrier transport of highly doped ZnO polycrystalline films. More study on the effects that the two-step deposition processes using CLs exert on the textured structure and properties of ZnO-based transparent conductive films as a function of total film thickness of less than 500 nm will be given elsewhere.

## Conclusions

In this letter, we have reported the development of a two-step deposition process: (1) 10-nm-thick CLs made from GZO films on glass substrates by IP with dc arc discharge and (2) subsequent deposition of 490-nm-thick AZO films on the CLs by DC-MS. This growth method at a substrate temperature of 200 °C was employed to produce AZO films exhibiting high *μ*_H_. The CLs having a texture in which the *c*-axis was preferentially oriented perpendicular to the substrate surface play a critical role in achieving AZO polycrystalline films having a textured structure with a well-defined single (0001) orientation. We have obtained the following experimental results: (1) a small FWHM of the *ω* rocking curves for the 0002 peak of 1.81°; (2) a high *μ*_H_ of 50.1 cm^2^/Vs with an *N* of 2.55 × 10^20^ cm^−3^, leading to a low *ρ* of 4.89 × 10^−4^ Ωcm. This study clearly shows that a technology to produce AZO films with a high degree of *c*-axis alignment between columnar grains is essential for enhancing intragrain carrier mobility together with little contribution of GB scattering to carrier transport. The development of the CL technique with a nanoscale materials design of very thin films is expected to help us understand the mechanisms of carrier transport of highly doped ZnO polycrystalline films.

## Abbreviations

AZO, Al-doped ZnO; BZO, B-doped ZnO; CL, critical layer; dc, direct current; FWHM, full width at half maximum; GB, grain boundary; GIXRD, grazing-incidence x-ray diffraction; GZO, Ga-doped ZnO; IP, ion plating; MS, magnetron sputtering; NIR, near-infrared; PLD, pulsed laser deposition; RF, radio frequency; RSM, reciprocal space maps; VIS, visible; XRD, x-ray diffraction
